# Female sexual dysfunction in anti-synthetase syndrome

**DOI:** 10.1590/1806-9282.20260486

**Published:** 2026-10-23

**Authors:** Lorenza Rosa Silvério da Silva, Theo Lerner, Elsa Aida Gay de Pereyra, Alexandre Moura dos Santos, Samuel Katsuyuki Shinjo

**Affiliations:** 1 Universidade de São Paulo, Faculdade de Medicina, Division of Rheumatology – São Paulo (SP), Brazil.; 2 Universidade de São Paulo, Faculdade de Medicina, Hospital das Clínicas, Division of Obstetrics and Gynecology – São Paulo (SP), Brazil.

**Keywords:** Antisynthetase syndrome, Idiopathic inflammatory myopathies, Sexual dysfunction, Physiological, Women, Cognitive behavioral therapy

## Abstract

**OBJECTIVE:**

Sexual dysfunction has not been investigated in women with anti-synthetase syndrome. The aim of this study was to determine the prevalence of sexual dysfunction in women with anti-synthetase syndrome compared with healthy controls (CTR), to evaluate associated clinical factors, and to assess the effect of cognitive behavioral therapy on sexual function and related outcomes.

**METHODS:**

This single-center study included a cross-sectional evaluation conducted between March and August 2023 and a prospective interventional phase. Thirty-three women with anti-synthetase syndrome and 31 CTR were included. Sexual function, sexual distress, quality of life, fatigue, depression, body image, physical activity, and disease-related parameters were assessed using validated questionnaires. Multivariate logistic regression was used to identify factors independently associated with sexual dysfunction. Patients with anti-synthetase syndrome and sexual dysfunction were invited to participate in group-based cognitive behavioral therapy, with up to 90 days’ follow-up.

**RESULTS:**

Groups were comparable with respect to age and marital status. Sexual dysfunction prevalence was significantly higher in the anti-synthetase syndrome group than in CTR (81.8 vs. 51.6%; p=0.016). Anti-synthetase syndrome was independently associated with sexual dysfunction (OR 4.11; 95%CI 1.20–15.78). Within the anti-synthetase syndrome cohort, patients with and without sexual dysfunction were similar across most variables, except for prednisone use, which occurred only in patients with sexual dysfunction. Six patients completed cognitive behavioral therapy, showing improvements in sexual distress, sexual function—particularly arousal—and fatigue at 90 days.

**CONCLUSION:**

Sexual dysfunction is highly prevalent in women with anti-synthetase syndrome and appears intrinsically associated with the disease. Cognitive behavioral therapy represents a promising non-pharmacological strategy to improve sexual function, distress, and fatigue in this population.

## INTRODUCTION

Anti-synthetase syndrome (ASyS) is a distinct subset of idiopathic inflammatory myopathies characterized by the presence of anti-aminoacyl-transfer-ribonucleic acid synthetase autoantibodies and a heterogeneous, multisystem clinical spectrum. Classically, ASyS is defined by the triad of inflammatory myopathy, arthritis, and interstitial lung disease; however, disease expression is highly variable and may evolve throughout the disease course. Additional manifestations such as fever, Raynaud’s phenomenon, and hyperkeratotic fissuring of the fingers, commonly referred to as “mechanic’s hands,” are frequently observed and may contribute to functional impairment, occupational limitations, and reduced quality of life. Anti-Jo-1 is the most prevalent anti-synthetase antibody, although several other autoantibodies have been described and may be associated with distinct clinical phenotypes and prognostic profiles^1^.

Sexual health is a fundamental component of global health and well-being, encompassing biological, psychological, interpersonal, and sociocultural dimensions. It extends beyond sexual activity itself and includes sexual identity, intimacy, emotional connection, self-esteem, and satisfaction. Sexual dysfunction (SD) is defined as persistent or recurrent disturbance in sexual desire, arousal, orgasm, or satisfaction that results in clinically significant distress or interpersonal difficulty^2,3,4^. These disturbances arise from complex interactions involving neuroendocrine regulation, vascular integrity, musculoskeletal function, emotional processing, cognitive factors, and contextual influences such as relationship dynamics and cultural background.

Systemic autoimmune rheumatic diseases (SARDs) are consistently linked with impaired sexual health. Chronic pain, fatigue, stiffness, physical disability, mucocutaneous dryness, altered body image, depressive and anxiety symptoms, hormonal dysregulation, and adverse effects of pharmacological therapies may all contribute to SD in this population^5,6,7^. In addition, uncertainty regarding disease prognosis, fear of symptom exacerbation during sexual activity, and changes in partner dynamics may further impair sexual well-being. Despite its high prevalence and substantial impact on quality of life, sexual health remains underrecognized and underdressed in routine rheumatologic care. Many patients report embarrassment, lack of opportunity, or discomfort in discussing sexual concerns with healthcare professionals, resulting in unmet needs and persistent distress^7^.

It is well established that multiple factors contribute to SD—such as medical conditions, medication use, psychological factors, and relationship-specific issues^8^. However, although it is known that these factors lead to reduced sexual activity, there are no specific pathophysiological mechanisms that fully explain the phenomenon in isolation; this supports the biopsychosocial hypothesis of female sexual function, which integrates neuroendocrinological, psychosocial, and personal factors.

Within idiopathic inflammatory myopathies, SD has been documented in both men and women and has been associated with muscle weakness, reduced physical activity, fatigue, depressive symptoms, and impaired quality of life^9^. Importantly, these associations appear to be largely independent of disease duration or type of immunosuppressive therapy, suggesting that SD may represent a core disease-related burden rather than a secondary consequence of treatment or chronicity. In ASyS specifically, interstitial lung disease and its associated dyspnea and reduced exercise tolerance, chronic glucocorticoid exposure, persistent fatigue, disease-related changes in body image, psychological distress, and systemic inflammatory activity have been proposed as plausible, disease-specific contributors to impaired sexual function, consistent with the biopsychosocial model of female sexual health endorsed by the International Society for the Study of Women’s Sexual Health and the International Consultation on Sexual Medicine^3,8^. However, to date, no study has specifically evaluated sexual function in patients with ASyS, a subgroup characterized by systemic involvement, pulmonary disease, long-term immunosuppression, and complex psychosocial challenges.

Given the absence of disease-specific data and the growing recognition of sexual health as an essential domain of patient-centered care, this study aimed to: (1) determine the prevalence of SD in adult female patients with ASyS compared with healthy controls; (2) identify demographic, clinical, laboratory, and psychosocial factors associated with SD; and (3) prospectively assess the impact of cognitive-behavioral therapy (CBT) on sexual function, sexual distress, and related outcomes in women with ASyS.

## METHODS

### Study design and participants

This single-center study consisted of a cross-sectional observational phase followed by a prospective interventional phase. Adult female patients with ASyS receiving regular follow-up at a tertiary rheumatology center were consecutively invited to participate between March and August 2023. ASyS was defined according to the criteria proposed by Behrens Pinto et al.^10^. Only patients with stable disease and regular outpatient follow-up were considered eligible to minimize confounding related to acute disease exacerbations.

Exclusion criteria included pregnancy; active disease requiring escalation of immunosuppressive therapy, high-dose glucocorticoids, intravenous immunoglobulin, or pulse therapy; overlap syndromes with other connective tissue diseases; history of gynecological malignancy or pelvic irradiation; and active gynecological infection at the time of evaluation. These criteria were applied to minimize potential bias related to conditions independently associated with SD.

A control group was composed of adult women without rheumatologic diseases who completed electronic questionnaires during the same period. Controls were recruited to provide a reference population for sexual function outcomes and were required to meet the same exclusion criteria related to gynecological conditions.

### Data collection and assessments

All participants completed standardized questionnaires addressing sociodemographic characteristics, educational level, socioeconomic status, marital and relationship status, gynecological history, comorbidities, lifestyle habits, and anthropometric data. Sexual function was assessed using validated Brazilian Portuguese versions of the Female Sexual Function Index (FSFI)^11^, the Female Sexual Distress Scale–Revised (FSDS-R)^12^, and the Female Sexual Quotient (FSQ)^13^. SD was defined as FSFI ≤26.55 and/or FSQ ≤60, while sexual distress was defined as FSDS-R ≥11.

Disease activity and functional status were evaluated using Manual Muscle Testing (MMT-8), Myositis Disease Activity Assessment Visual Analog Scales (MYOACT), and the Health Assessment Questionnaire (HAQ). Physician and patient-reported global assessments were obtained using visual analog scales to capture both clinical and subjective perceptions of disease impact. Fatigue was assessed using the Fatigue Severity Scale, depressive symptoms using the Beck Depression Inventory, body image dissatisfaction using the Body Shape Questionnaire, physical activity using the International Physical Activity Questionnaire–Short Form, pain using a visual analog scale, and quality of life using the EQ-5D questionnaire^14,15,16^.

Laboratory parameters, including serum muscle enzymes and myositis-specific and myositis-associated autoantibody profiles, as well as current and prior pharmacological treatments, were obtained from medical records and reviewed by a single investigator to ensure data consistency^17^.

### Prospective intervention

Patients with ASyS who met criteria for SD were invited to participate in a group-based CBT intervention. Sessions were conducted weekly over a 12-week period and followed a previously validated semi-structured protocol^18^. The intervention focused on increasing sexual self-awareness, addressing dysfunctional beliefs related to sexuality, reducing performance anxiety, improving communication skills, and promoting adaptive coping strategies. Sexual function questionnaires were administered before the intervention and at 30 and 90 days after completion to assess short and medium-term effects.

### Statistical analysis

Continuous variables were expressed as means±standard deviation or medians with interquartile ranges, as appropriate. Categorical variables were presented as absolute and relative frequencies. The normality of continuous variables was assessed using the Shapiro-Wilk test. Normally distributed continuous variables were compared between groups using Student’s t-test, whereas non-normally distributed variables were compared using the Mann-Whitney U test; categorical variables were compared using the chi-square test or Fisher’s exact test, as appropriate. Group comparisons were performed using parametric or nonparametric tests according to data distribution. Multivariate logistic regression analysis was applied to identify factors independently associated with SD. Candidate variables for inclusion in the multivariate model were selected based on their statistically significant association with SD in univariate analysis (p<0.05); the study group (ASyS vs. controls) was retained in the model as the primary variable of interest, given the central hypothesis of the study. Missing data were not imputed; participants with missing values for a given variable were excluded from the analyses involving that specific variable (complete-case analysis). Statistical significance was set at p<0.05. Statistical power analysis and computational procedures followed established methodological standards^19,20^. Given the limited sample size, particularly in the prospective interventional phase, inferential results from that phase should be interpreted with caution and regarded as exploratory and hypothesis-generating rather than confirmatory.

## RESULTS

### Cross-sectional analysis

A total of 33 women with ASyS and 31 healthy controls were included in the analysis (Figure 1). Groups were comparable regarding age and marital status. Patients with ASyS presented lower educational attainment, poorer socioeconomic status, and higher body mass index compared with controls.

**Figure 1:**
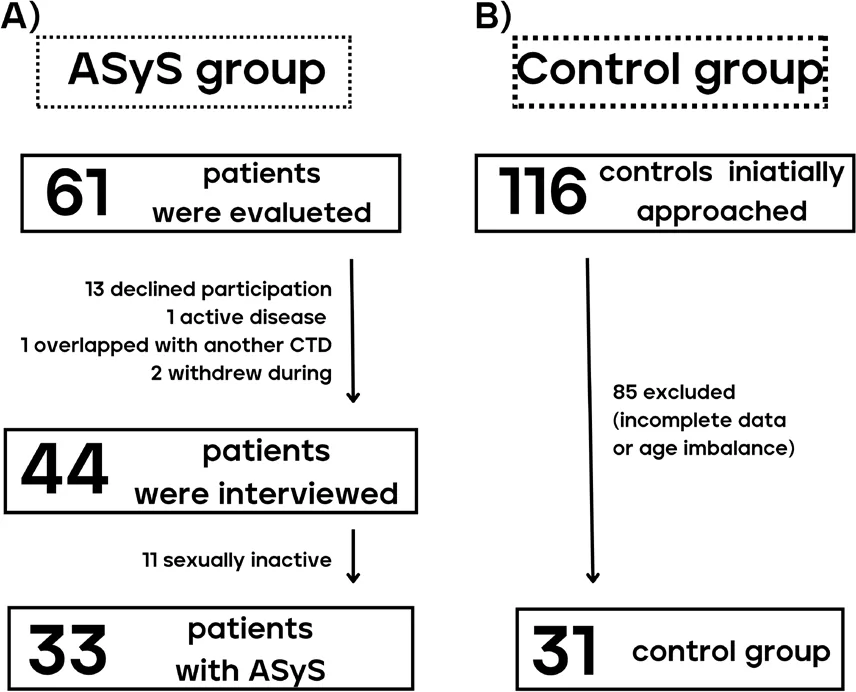
Flowchart of participant selection. (A) Selection of participants with anti-synthetase syndrome. (B) Selection of participants in the control group. ASyS: anti-synthetase syndrome; CTD: connective tissue disease.

r therapy on sexual dysfunction of women in reproductive age: a systematic review. Int J Sex Health. 2024;36:287-301. https://doi.org/10.1080/19317611.2024.2360731

The prevalence of SD was significantly higher in the ASyS group than in controls (81.8 vs. 51.6%; p=0.016). In multivariate analysis, ASyS was independently associated with SD (OR 4.11; 95%CI 1.20–15.78). Candidate variables entered into the multivariate model were median age, race, economic status, educational level, and body mass index, all of which showed a statistically significant association with SD in univariate analysis (p<0.05); no candidate variable was excluded from the model. After adjustment, ASyS remained independently and significantly associated with SD, whereas none of the other covariates retained a significant association with SD (OR>1 for all; p>0.50 for all).

Within the ASyS cohort, patients with and without SD did not differ significantly in terms of disease activity scores, laboratory parameters, autoantibody profiles, fatigue, depressive symptoms, pain, body image dissatisfaction, quality of life, or physical activity. Prednisone use was observed exclusively among patients with SD.

### Prospective analysis

All ASyS patients with SD were invited to participate in CBT. Eight patients initially enrolled, and six completed the intervention (Table 1). At 90-day follow-up, significant improvements were observed in sexual distress and sexual function scores, with particular improvement in the arousal domain. Fatigue scores also decreased after the intervention, while disease activity and other psychosocial measures remained unchanged.

**Table 1:** General characteristics of patients with anti-synthetase syndrome and sexual dysfunction with behavioral therapy intervention.

Parameters	Baseline	D90	p-value
Disease status			
HAQ (0.00–3.00)	0.13 (0.00–0.72)	0.50 (0.00–1.00)	0.789
MMT-8 (0–80)	80 (78–80)	78 (76–78)	0.169
MYOACT	0.0 (0.0–0.2)	0.1 (0.0–0.2)	0.427
PGA (0–10)	6.0 (1.3–7.0)	6.0 (1.5–6.0)	0.587
PhGA (0–10)	0.0 (0.0–3.8)	0.0 (0.0–2.4)	0.346
Laboratory data			
CPK (U/L)	182 (80–396)	258 (137–398)	0.562
LDH (U/L)	234 (198–239)	238 (212–250)	>0.999
AST (U/L)	22 (18–24)	27 (25–36)	0.312
ALT (U/L)	20 (17–21)	22 (19–35)	0.343
BSQ (34–204)	83 (69.2–106)	66 (46.2–84.2)	0.177
BDI (0–63)	15 (12.5–19.8)	16 (11.2–17)	0.833
Pain	2 (33.3)	5 (83.3)	0.242
PGA EQ5D (0–100)	80 (53–100)	100 (100–100)	>0.999
IPAQ-SF			
Metabolic equivalent (MET/week)	2,050 (1,006–3,452)	2,876 (1,238–4,798)	0.844
Sedentary behavior (hours/day)	3.0 (2.1–5.8)	2.7 (2.0–4.6)	>0.999

Data are expressed as median (interquartile range) or frequency (%). ALT: alanine aminotransferase; AST: aspartate aminotransferase; ASyS: anti-synthetase syndrome; BDI: Beck Depression Inventory; BSQ: Body Shape Questionnaire; CPK: creatine phosphokinase; EQ5D: Quality of Life Questionnaire by EuroQol; FSS: Fatigue Severity Scale; HAQ: Health Assessment Questionnaire; IPAQ-SF: International Physical Activity Questionnaire—Short Form; LDH: lactate dehydrogenase; MET: metabolic equivalent; MMT: Manual Muscle Testing; MYOACT: Myositis Disease Activity Assessment Visual Analog Scale; PGA: Patient Global Assessment; PhGA: Physician Global assessment; SD: sexual dysfunction.

## DISCUSSION

In this study, SD was highly prevalent among women with ASyS, exceeding that observed in healthy controls and aligning with rates reported in other SARDs^2,5,7^. Importantly, SD was independently associated with the diagnosis of ASyS, rather than with disease activity, laboratory markers, or psychosocial variables, suggesting that SD may represent an intrinsic and underrecognized burden of the disease. The prevalence of SD in the control group (51.6%) aligns with the global prevalence of 50.8% reported by Alidost et al^21^.

The absence of associations between SD and fatigue, depression, or body image contrasts with findings reported in other rheumatic diseases and may reflect unique pathophysiological mechanisms or psychosocial dynamics specific to ASyS. Chronic pulmonary involvement, fluctuating disease course, and long-term exposure to glucocorticoids may contribute to altered sexual function through complex biological and psychological pathways. Although prednisone use was observed exclusively among patients with SD, causal inference is limited by the cross-sectional design. Further investigation is warranted, and future multicenter, longitudinal studies may help clarify this issue.

Prospective findings suggest a potential benefit of CBT, which may be a potentially effective non-pharmacological intervention for improving sexual function and reducing sexual distress in women with ASyS. These findings are considered exploratory and hypothesis-generating, requiring validation in larger controlled prospective studies. Improvements were observed particularly in domains related to sexual arousal and distress, consistent with previous studies demonstrating the efficacy of CBT and mindfulness-based approaches in female SD^22,23,24^. Addressing sexual health through structured psychological interventions may therefore represent a valuable adjunct to standard rheumatologic care, particularly in conditions for which pharmacological options are limited.

Several limitations should be acknowledged. The single-center design and relatively small sample size may limit generalizability. In addition, the absence of a control group in the interventional phase precludes definitive conclusions regarding treatment efficacy. Nevertheless, this study represents the first investigation specifically focused on sexual health in ASyS and provides a foundation for future research. It is important to highlight that, given the high prevalence of SD in patients with ASyS and its direct impact on quality of life and, potentially, on adherence to treatment, routine screening for sexual health should be encouraged, as should the involvement of a multidisciplinary team (rheumatologists, pelvic floor physical therapists, psychologists, and sexual medicine specialists).

## CONCLUSION

SD is highly prevalent in women with ASyS and appears to be intrinsically associated with ASyS, independent of disease activity or most psychosocial factors. The findings underscore the importance of systematically addressing sexual health in the clinical management of ASyS. Cognitive-behavioral therapy may improve sexual function, reduce sexual distress, and alleviate fatigue, representing a promising adjunctive strategy. Larger, multicenter, controlled studies are warranted to confirm these findings and guide evidence-based clinical interventions.

## Data Availability

The datasets generated and/or analyzed during the current study are available from the corresponding author upon reasonable request.
